# Cancer Precision Medicine in China

**DOI:** 10.1016/j.gpb.2016.10.002

**Published:** 2016-10-14

**Authors:** Hui Li

**Affiliations:** 1 National Clinical Research Center for Cancer (Tianjin), Tianjin 300060, China; 2 Department of Gastrointestinal Cancer Biology, Tianjin Medical University Cancer Institute & Hospital, Tianjin 300060, China

## Background

Cancer, as a global health issue, has been attracting increasing attention from scientists, medical professionals, as well as the general population. During the past several decades, many therapeutic approaches, including surgery, chemotherapy, radiotherapy and immunotherapy, have come into play for cancer treatment. Nonetheless, many of these treatments are not personalized according to the specific situation or genetic context of a particular patient. Nowadays, precision medicine, defined by President Obama as “delivering the right treatments at the right time—every time—to the right person”, opens a new era of cancer research with the hope to provide personalized treatment for different patients. To foster the knowledge share and promote potential collaboration on cancer precision medicine domestically and internationally, the first International Conference on Cancer Precision Medicine (IConCPM) took place on September 22–24, 2016 in Tianjin, China. The conference was hosted by the National Clinical Research Center for Cancer (NCRCC), Chinese Anti-Cancer Association (CACA), China Medicinal Biotechnology Association (CMBA), Medical and Health Engineering Division of Chinese Academy of Engineering (CAE), Tisch Cancer Institute at the Icahn School of Medicine at Mount Sinai, as well as the National Foundation of Cancer Research (NFCR). IConCPM was initiated by Prof. **Xishan Hao**, CAE academician from NCRCC and Prof. **Raju Kucherlapati**, member of the National Academy of Science of the United States (US) from Harvard Medical School. As the conference chair, Prof. **Hao**, together with co-chair Prof. **Kucherlapati**, delivered a warm welcome speech. More than 300 people from all over the world attended this conference and 23 talks were presented at this meeting, focused on precision medicine in China. Then Prof. **Ping Wang**, President of Tianjin Medical University Cancer Institute and Hospital, and **Zhaofeng Zhang**, Director, Division of Biotechnology and Medicine, Department of Science and Technology for Social Development, Ministry of Science and Technology of China, also gave their brief remarks for welcome. The conference was preceded by a next-generation sequencing (NGS) and bioinformatics analysis workshop on Sept 22, covering topics from DNA/RNA extraction to sequencing data analysis. In this report, we provided a short introduction on the talks and briefly recapitulated each one of them referring to related publications whenever possible. At the end, we summarize this report with a few comments on the success of IConCPM.

## Topics

In the morning session on Sept 23, three academicians from CAE or Chinese Academy of Sciences (CAS), China, and one member of the National Academy of Science, US, presented their work on precision medicine. Prof. **Hao** overviewed the current status of cancer care and precision medicine projects in China, touching global burden of cancer, status of cancer prevention and control, precision medicine and clinical applications in cancer precision medicine. NCRCC, under his leadership, has been committed to establishing precision medicine research platform which includes cancer tissue bank, clinical trial unit, cancer molecular diagnosis, and bioinformatics core. This platform will offer risk prediction, early diagnosis, target therapy, prognosis evaluation, and drug development to help realize the power of precision medicine. Next, Prof. **Webster Cavenee** from the Ludwig Institute for Cancer Research emphasized in his talk the necessity of international cooperation in cancer clinical trials for precision medicine. One single traditional clinical trial would need hundreds to thousands of patients, but it may turn out impossible to recruit enough patients for certain diseases. Take the US as an example, there are simply not enough patients, say for glioblastoma (GBM), in the entire country to do more than an extremely small number of clinical trials, not even to mention stratification on the basis of ethnicity or other factors. In this context, GBM-AGILE (Adaptive Global Innovative Learning Environment) was born to address these issues, which has to accrue needed numbers of patients for combination trials. The goals of GBM-AGILE are rapid and efficient testing of therapeutic agents and their combinations, as well as developing databases of clinical trials, biomarkers, and molecular data. This global platform will bring frontline trials to a consortium of countries, providing sufficient patients to stratify and test drug combinations. Afterward, Prof. **Yixin Zeng** from the Beijing Hospital focused his talk on molecular classification and personalized medicine. How does molecular classification lead to precision medicine? The answer is to find the right patients, give the right drug at the right timing. Prof. **Zeng** took the example of nasal pharyngeal cancer (NPC) to illustrate the importance of personalized prevention strategy and molecular classification, which mainly refers to immune markers including immune cell sub-typing, cytokines, major histocompatibility complex (MHC) I and II molecules, B7 family and immune-related inhibitors. He concluded that precision medicine, on the basis of molecular classification, will lead to future development of clinical practice and make modern medicine more cost-effective. In the following talk, Prof. **Qimin Zhan** from the Peking University Health Science Center introduced his research on genomic alterations of esophageal squamous cell carcinoma (ESCC) [1]. Prof. **Zhan** and his group identified 8 significantly-mutated genes in ESCC and mutations in the gene encoding family with sequence similarity 135 member B (*FAM135B*) are correlated with clinical characteristics. They also found miR-548k, a microRNA (miRNA) that was amplified and overexpressed in ESCC, possesses oncogenic characteristics *in vivo* and *in vitro*. In addition, genetic alterations are also revealed in genes encoding components of multiple pathways including Wnt pathway, Notch pathway, RTK-Ras, and Akt pathways. Their findings support the notion that cancer is now considered fundamentally as a disease of genomic alteration. After the four presentations, there was **a panel discussion – Roles of technology, data and analytics in precision medicine**. Dr. **Lynda Chin** from Institute for Health Transformation, University of Texas System and Dr. **Peter Bahrs** from IBM Watson Health Implementations Team co-chaired this section and discussed the roles of technology, cognitive analytics, and public–private partnerships in precision medicine.

In the afternoon session on the first day (Sept 23), 11 speakers were invited to share their insightful opinions or brilliant work. Prof. **Kucherlapati** summarized the Precision Medicine Initiative of the US, featured by disease prevention, detection, and treatment through the precise manner. He also introduced the Moon Shots Program with the ultimate goal to end the threats of cancer. This program was launched by M. D. Anderson Cancer Center in 2012. On January 12, 2016, President Obama announced a Cancer Moonshot initiative to accelerate cancer research, which aimed to make more therapies available to more patients and improve our ability to prevent cancer and detect it at an early stage. Next **Qing Li**, Director, Science and Development Centre, the National Health and Family Planning Commission of China, introduced the reform of science and technology program management system and the foundation of the several national-level projects. And in the end, he shared his view on the China Precision Medicine Initiative. Then Prof. **Daniel Sulivan** from the Moffitt Cancer Center presented their practice of the precision medicine in the Oncology Research Information Exchange Network (ORIEN), which is a network of several major cancer centers in the US, with ultimate goals to provide evidence for decision-making and clinical care. Following these talks was the immunotherapy and chimeric antigen receptor T-cell (CAR-T) session. First, Prof. **Steven Burakoff** from Mount Sinai School of Medicine presented their work of identifying hematopoietic progenitor kinase 1 (HPK1) as a novel immunotherapy target [2]. Next Prof. **Renier J. Brentjens** from the Memorial Sloan Kettering Cancer Center, who leads one of the first teams conducting CAR-T therapy in the world, introduced their latest clinical trials on several cancers with diverse modifications of the therapeutic cells [3], [4], [5], [6], [7], [8]. After the tea break, Prof. **Cheng Qian** from the Third Military Medical University shared their clinical practice with modified dendritic cells (DCs) and CAR-T on several cancer types. Then Prof. **Chunrong Tong** from the Lu Daopei Hematology Oncology Center introduced their experience on utilization of anti-CD19 CAR-T cells for refractory/relapsed b acute lymphoblastic leukemia. Next as a collaborator of Prof. Tong, Prof. **Hongsheng Zhang** from the Tongji University School of Medicine presented their translational research and clinical application of CAR-T therapy. Prof. **Weidong Han** from the Chinese PLA General Hospital then summarized their clinical practice of CAR-T cell therapy on both hematologic cancer and solid tumor [9], [10], [11]. Prof. **Zhong Li** from the Second Military Medical University talked about his perspective on cancer immunotherapy. At last, Prof. **Xiubao Ren** from the Tianjin Medical University Cancer Institute and Hospital summarized the overall clinical progress of CAR-T cells in cancer treatment.

In the morning of Sept 24, Dr. **Ying Wang**, Secretary General of CACA, and Dr. **Michael Wang** from NFCR both introduced the contribution of academic societies in precision medicine. In her presentation, Dr. **Wang** introduced CACA, the role of CACA in cancer prevention and control, as well as the role of CACA in cancer precision medicine. Then Prof. **Bruce Johnson** from the Harvard Medical School and Dana Farber Cancer Institute gave his presentation about the impact of genomic changes on precision medicine for lung cancer [12]. Mutations in the gene encoding epithelial growth factor receptor (EGFR), rearrangements of the gene encoding anaplastic lymphoma kinase (ALK), and other oncogenic drivers, such as *BRAF*, are the most common genomic changes in lung cancer. The Lung Cancer Mutation Consortium (LCMC) is planning analyses on these genomic changes and will establish a virtual database that may help doctors and researchers determine the frequency of certain mutations and explore opportunities for clinical trial enrollment. Prof. **Toshifumi Wakai** from Niigata University next introduced the genomic sequencing for optimal cancer treatment in gastrointestinal cancer. He and his group demonstrated concordance of CancerPlex with whole-exome sequencing from the TCGA in identifying hypermutated samples and microsatellite instability in multiple cancer types, such as colorectal and gastric cancers. They also highlighted the clinical utility of CancerPlex in guiding treatment strategies with targeted therapy in solid tumors, thus demonstrating how to harness the power of NGS in actualizing precision medicine. Prof. **Mu-Sheng Zeng** from Sun Yat-sen University presented his work on advances of basic and translational study of NPC. They found that expression of B lymphoma Moloney murine leukemia virus insertion region 1 homolog (Bmi-1) or human telomerase reverse transcriptase (hTERT), and knockdown of p16 enabled immortalization of nasopharyngeal epithelial cells (NPECs). Sphere-like culture of *Bmi-1* immortalized cells supported high efficiency of cell-free infection of Epstein–Barr virus (EBV), an oncovirus for NPC. They also demonstrated that RNA binding motif protein 24 (RBM24) played a role in NPC by modulating the stability of miRNAs and long non-coding RNAs. In the next talk, Prof. **Andreas Keller** from Saarland University focused on miRNAs and cancer. They found that blood-borne miRNAs were stable and powerful biomarkers for early diagnosis and companion diagnostics in cancer that are currently pushed from research to clinics. He also cautioned the biases present when measuring microRNA expression from different sources or using different platforms. These issues should be well appreciated and taken into account for translation into clinical practice. After the talk, there was a panel discussion co-chaired by Prof. **Qiang Li** from Tianjin Medical University Cancer Institute and Hospital and Prof. **Xiaoming Zou** from EOC Pharma, discussing the target therapies and drug development in the era of precision medicine.

In the afternoon session on the second day (Sept 24), the meeting started with two panel discussions about genetic testing for cancer and investments in precision medicine in China which were chaired by **Tuan Ha-Ngoc** from KEW and **Tom Miller** from Greybird Ventures, respectively. In the following session chaired by Prof. Keller and myself, **Zhimin Yang** from Center for Drug Evaluation, China Food and Drug Administration, talked about issues that should be considered for development and evaluation of oncology products in the perspective of precision medicine. She illustrated her viewpoint on the model evolution of oncology product development, precision medicine, development of clinical innovation products, as well as the considerations for development and evaluation under the new model for precision medicine. Prof. **Yi Zhao** from Institute of Precision Medicine, CAS reported his work on big data platform construction under the China Precision Medicine Initiative, aimed to build the most complete genetic library of the Chinese population. The conference ended with a panel discussion co-chaired by Prof. **Hao** and Prof. **Kucherlapati**. Featuring the future of precision medicine in China, participants provided their comments to questions including (1) what technologies are for precision medicine; (2) how these technologies can impact the patient treatment today; (3) how to identify the right patients for new trials; (4) who are going to pay; and (5) what China can benefit from the lessons learnt in precision medicine projects in the US.

## Conclusion

The conference ended successfully and has received numerous positive feedbacks from the participants. The conference provided a perfect chance for overseas and domestic scientists and physicians to communicate and share their understanding and practice of the Precision Medicine. Meanwhile the conference provided a productive platform for elite from the related fields to collaborate from decision making to drug development and investment. Other than the informative talks and insightful discussion, the conference organizers also made kind arrangements in having slides in both English and Chinese simultaneously with the live interpretation, making sure smooth communication.

Given the emerging need of precision medicine in China, the conference introduced overseas system and practice of precision medicine into China as examples. Involvement of people with diverse roles in precision medicine from doctors, researchers, policy makers, industrialists to investors altogether would help to find answers or address specific concerns for China as raised during the conference, and hopefully result in a wider and deeper collaboration among different parties along the whole process of precision medicine for cancer clinical care.
